# Cross-Leg Free Flap: Crossing the Border Zone of Ischemic Limb—A Case Report of Limb Salvage Procedure following a Delayed Diagnosis of Popliteal Artery Injury

**DOI:** 10.1055/a-1962-6009

**Published:** 2023-02-07

**Authors:** Hui Yuan Lam, Wan Azman Wan Sulaiman, Wan Faisham Wan Ismail, Ahmad Sukari Halim

**Affiliations:** 1 Hospital Universiti Sains Malaysia, Medical Campus, Universiti Sains Malaysia, Kota Bharu, Kelantan, Malaysia; 2 Reconstructive Sciences Unit, School of Medical Sciences, Medical Campus. Universiti Sains Malaysia, Kota Bharu, Kelantan, Malaysia; 3 Orthopaedic Oncology and Reconstructive Unit (OORU), School of Medical Sciences, Medical Campus. Universiti Sains Malaysia, Kota Bharu, Kelantan, Malaysia

**Keywords:** popliteal artery, free flap, lower limb, knee dislocation

## Abstract

Vascular injury following traumatic knee injury quoted in the literature ranges from 3.3 to 65%, depending on the magnitude and pattern of the injury. Timely recognition is crucial to ensure the revascularization is done within 6 to 8 hours from the time of injury to avoid significant morbidity, amputation, and medicolegal ramifications. We present a case of an ischemic limb following delayed diagnosis of popliteal artery injury after knee dislocation. Even though we have successfully repaired the popliteal artery, the evolving ischemia over the distal limb poses a reconstruction challenge. Multiple surgical debridement procedures were performed to control the local tissue infection. Free tissue transfer with chimeric latissimus dorsi flap was done to resurface the defect. However, the forefoot became gangrenous despite a free muscle flap transfer. His limb appeared destined for amputation in the vicinity of tissue and recipient vessels, but we chose to use a cross-leg free flap as an option for limb salvage.

## Introduction

Popliteal artery injury in the form of transaction, blockage, laceration, perforation, arteriovenous fistula, or an intimal injury after blunt trauma to the lower extremity has been shown to range from 28 to 46%. 1 2 3 The popliteal artery injury is frequently associated with knee dislocation due to its unique anatomical relation to the muscle and bone. This restricts the motion of the popliteal artery, mainly when the tibia is displaced relative to the femur, resulting in traction or severe damage to its fixed parts. The popliteal artery gives off five genicular arteries within the popliteal space, but the collateral circulation around the knee is still lacking. Hence, popliteal injury is detrimental to limb perfusion, with the risk of amputation rate of 86% if the ischemic time is beyond 6 to 8 hours. 4

## Case

**Fig. 1:**
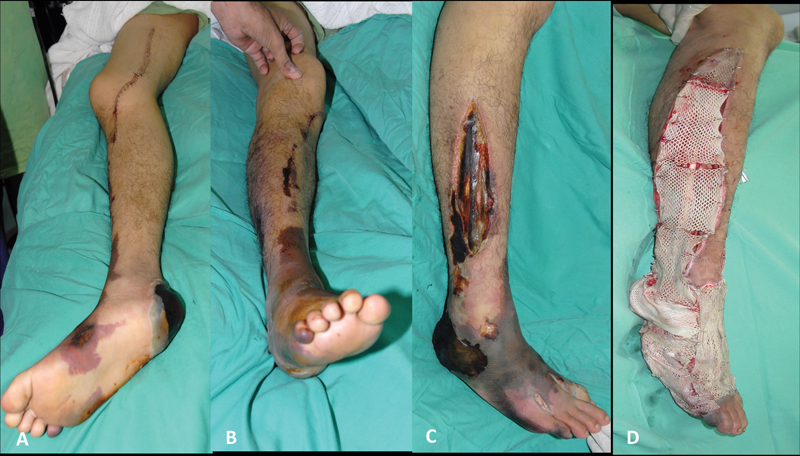
( **A, B** ) The limb was ischemic upon arrival to our center for revascularization. ( **C** ) Progressive ischemic over the soft tissue and muscle 3 days after revascularization and hyperbaric oxygen therapy. ( **D** ) Wound bed preparation with serial debridement and skin allograft application.

**Fig. 2:**
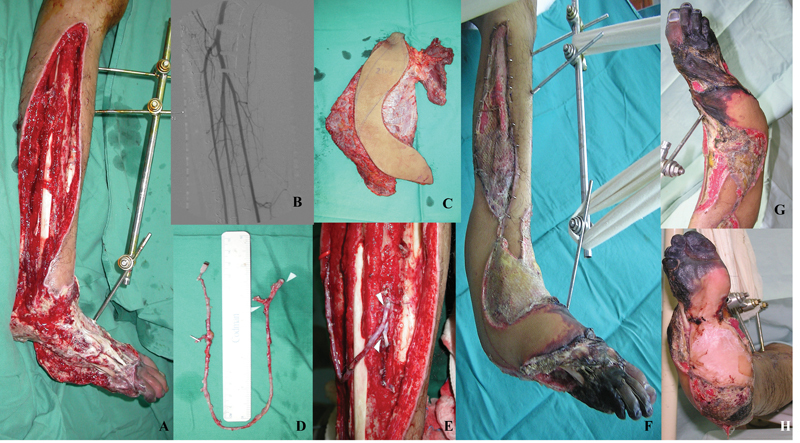
( **A** ) Pre- and post-lower limb reconstruction with chimeric LD-SA flap. ( **B** ) Serial surgical debridement was done till the wound bed is ready for soft tissue coverage with free flap. ( **C** ) Preoperative CT angiogram revealed patent bypass vein graft at the popliteal artery with well-opacified anterior tibial artery, posterior tibial artery, and perineal artery. ( **D, E** ) The chimeric LD-SA flap. AV-loop harvest by utilizing long saphenous vein from contralateral limb. ( **F–H** ) The two arrows showing the T-junction used for anastomosing the proximal and distal segment of recipient artery. The gangrenous forefoot with exposed over critical structures. The chimeric LD-SA flap survived well except for the necrosis at the distal end of the flap.

**Fig. 3:**
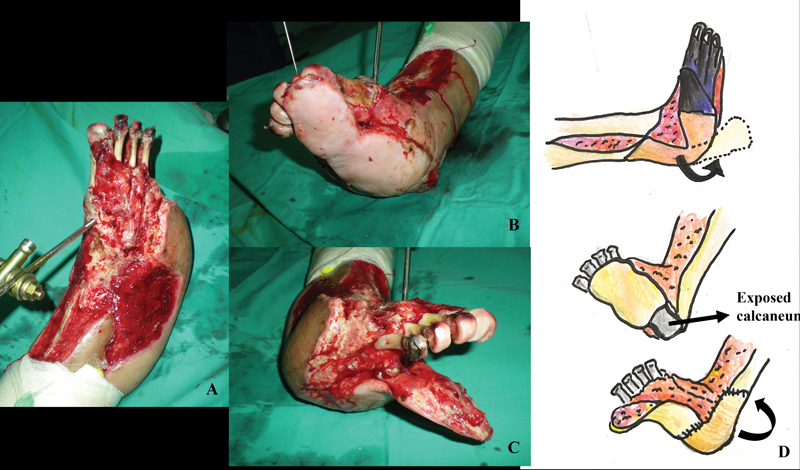
( **A** ) Reconstruction of the plantar region during the second free flap surgery. ( **B** ) Wound defect over the dorsum aspect of foot after transmetatarsal amputation of the distal phalanges and debridement over the distal part of latissimus dorsi flap. ( **C, D** ) The plantar tissue was viable. The Latissimus flap was released and rotated to cover the exposed calcaneum.

**Fig. 4:**
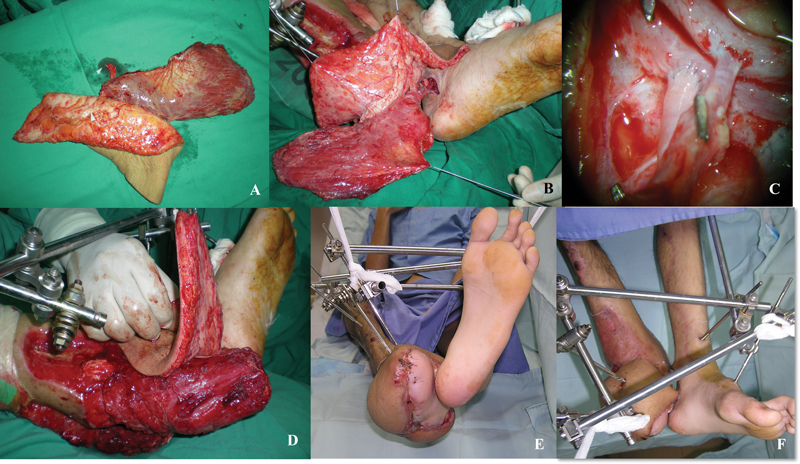
( **A** ) Second free flap surgery. ( **B, C** ) Chimeric ALT-VL flap. ( **D** ) End-to-side anastomosis of the chimeric ALT-VL free flap to posterior tibial vessels in the contralateral limb. ( **E,F** ) Vastus lateralis inset to obliterate the cavity over the lateral aspect of the foot and the remaining phalanges while the fasciocutaneous flap mobilized to cover the dorsum aspect of foot with part of the skin tabularized to protect the pedicle. The external fixation maintains the cross-leg free flap's position while enabling physiotherapy for both the knee and hip joints at the same time.

A 19-year-old gentleman had a motor vehicle accident resulting in a closed fracture of the medial condyle of the right tibia with posterior knee dislocation. He was initially treated in a district hospital with a backslab and underwent open reduction and plating of the right medial condyle. However, the heel and dorsum aspect of the foot became dusky postoperatively. Angiogram confirmed the suspected diagnosis of popliteal artery occlusion. He has then been referred to the nearest tertiary center for further management. The right femoral artery was explored after 1 week of the injury. Intraoperative findings showed the occlusion of a right popliteal artery which was consistent with the mechanism of injury. Without further delay, this case was referred to our center for revascularization. The assessment of his lower limb showed the presence of hard signs in acute limb ischemia ( Fig. 1 ). An expedited transfer to the operating room was done to explore and salvage the popliteal artery. There was a 9 cm thrombosed segment of the right popliteal artery, which required a long saphenous vein graft to restore the circulation of the distal limb. Upon completion of the anastomosis, all the distal pulses were palpable. Fasciotomy was performed to prevent further harmful necrosis since the total ischemic time from injury was approximately one week. He had undergone three cycles of hyperbaric oxygen therapy as an adjunct to improve the distal limb circulation. However, the muscles in the lateral compartment and the dorsum aspect of the foot became gangrenous. Serial surgical debridement was done until healthy and viable tissue was visible ( Fig. 2A ). CT angiography demonstrated a patent popliteal vein graft with well opacified anterior tibial artery, posterior tibial artery, and peroneal artery. Three weeks after the popliteal artery reconstruction, we decided to perform soft tissue coverage with chimeric latissimus dorsi – serratus anterior free flap to resurface the defect ( Fig. 2B ). We employed a single-stage AV loop for free flap connection as the short saphenous vein was thrombosed distally. Contralateral long saphenous vein harvested for AV loop construction with a T junction end used to anastomosis to the proximal and distal end of the anterior tibial artery. Unfortunately, the distal limb of the T junction of the donor vein is thrombosed intraoperatively, which results in end-to-end anastomosis to the proximal part of the anterior tibial artery ( Fig. 2C–E ). The free flap survived well, but the forefoot became gangrenous. The plantar tissue was still viable, and the remaining bone was healthy. We decided to salvage his limb with a cross-leg free flap with a chimeric anterolateral thigh—vastus lateralis muscle flap to cover the critical structure after debridement and trans metatarsal amputation ( Fig. 3 ). An additional layer of protection for the anastomosis was provided by tabularized part of the skin flap to the anastomosis site on the contralateral leg ( Fig. 4 ). This enables tension-free placement of the flap with a comfortable locking space such that both knees are relaxed and have a free range of motion. We had done flap division 6 weeks postoperatively, and the flap survived well. Six months postoperatively, no ischemic event or noticeable skin changes were observed. He can ambulate independently and is still under our annual follow-up to do secondary debulking surgery.

## Discussion

A traumatic lower limb associated with soft tissue damage, bone defect, vascular injuries with ischemic limb, and compartment syndrome is highly associated with amputation. 5 Lower limb trauma with artery injury has the most significant amputation rate, reported as high as 70%. 6 Meta-analysis by Perkins et al advocates that the anatomical level of arterial injury is one factor that influences limb salvage outcomes. 7 The level of artery injuries: femoral artery (upper zone), popliteal artery (middle zone), and anterior and posterior tibial artery (lower zone) do influence the revascularization outcomes. In addition, popliteal artery injury is proven to be catastrophic for amputation due to its limited collaterals to compensate during the prolonged ischemic time.

Traumatic artery injury can result in two amputations: primary and secondary amputation. Primary amputation was performed when there was no attempt for revascularization. The golden time of revascularization was 6 to 8 hours. Lower limbs with associated artery injuries could survive longer than amputated limbs because of the collateral circulation. However, a limb with a prolonged ischemic time is associated with a four-fold risk of secondary amputation. 8 The conditions prone to secondary amputations are local or systemic progressive infection and irreparable soft tissue or bone defects after debridement. So far, no literature has described salvage surgery to prevent primary and secondary amputation for a long-standing ischemic lower limb. Successful limb salvage with revascularization but with outcomes of a non-functional extremity do not improve the quality of life. A patient with optimum health status and young age reinforced the need to reconstruct the seemingly “unreconstructible” ischemic limb.

Timely diagnosis and vessel repair are the keys to a limb outcome. However, prolonged hypoperfusion caused by the initial injury is usually exacerbated by delayed revascularization, resulting in skeletal muscle ischemia that exceeds the permissible warm ischemic period. The contributing factors are technical and medical limitations, delay in diagnosis at local primary hospitals, preoperative resuscitation and evaluation, debridement of devitalized soft tissue, and patient transfer from one hospital to a tertiary center. 9 The average lower limb ischemia period in our center was 12.5 hours. This is due to the geographical problem, as it takes more than 8 hours to get from the district hospital to our hospital. A retrospective data collection from our patients (January 2018–June 2018) who have revascularized the lower limb after 6 hours of ischemia shows that only 6 out of 59 patients with acute kidney failure require hemodialysis. This is also consistent with our patient, who has a transient raised in the creatine kinase but no kidney failure that requires hemodialysis throughout the hospitalization. Although the delay is beyond our control, it is our philosophy to attempt revascularization and reserve primary amputation when it is life-threatening.

The ischemic-reperfusion injury comes at the cost of local and systemic consequences such as reperfusion injury, soft tissue, ischemic muscle necrosis, and fasciotomy wound infections. Failure to rescue from these complications imposes the risk of secondary amputations. In our case, we managed to control the progressive vascular zone of injury at the popliteal artery by using a bypass vein graft. However, we anticipate the distal limb's evolving ischemia in this case due to the long-standing ischemia before revascularization. Therefore, aggressive surgical debridement wound bed preparation and antibiotic usage are of prime importance to ensure surgical site sterilization before the first flap surgery. Once achieved, an integral part of the limb salvage process is planning proper recipient vessels in these large tissue defects with questionable vascular perfusion for microsurgery flap connection. Arteriogram was performed preoperatively, which showed the distal runoff included three vessels. Combined AV loop and chimeric Latissimus dorsi-serratus anterior free flap, at its core, navigate out of the zone of injury by creating a new conduit for anastomosis and extending the reach of pedicle for the inset of flap at the foot. The arterial and venous limb of the loop was connected in an end-to-end fashion to the corresponding flap vessels. Single-stage AV loop construction was contemplated as it is a single-stage surgery, and the tendency of the steal phenomenon is minimal in this young patient. A recent meta-analysis also advocates single-stage approach as thrombotic events and overall flap failures were significant in the two-stage approach. ^12^ However, there was a progression of ischemic over the distal part of the foot despite patency of AV loop and survived free flap. The thrombosis could be attributed to the T-junction of the AV loop's arterial limb, which affects the perfusion to the distal part of the anterior tibial artery. Keeping in mind the ischemic reperfusion insult to the distal limb in our case, this contributed to a double hit to the pedal arch patency and the angiosome encompassing the dorsum of the foot.

Given there is no autogenous conduit and distal location of the outflow, a pedal bypass using a distal vein patch or arteriovenous fistula was unlikely to work. Furthermore, even when the bone is healthy, widespread soft-tissue damage caused by ischemia complications usually compels the surgeon to do a higher-level amputation. However, we believed the foot might be salvaged since (1) the plantar tissue was healthy and sensate and (2) the presence of a healthy posterior compartment of muscle prevents foot deformity, limping, and limited joint movement. This is also reinforced by Moini et al, 10 who reported that in the presence of viable gastrocnemius muscle after popliteal artery injury, good functional outcomes of revascularization may be achieved.

**Fig. 5:**
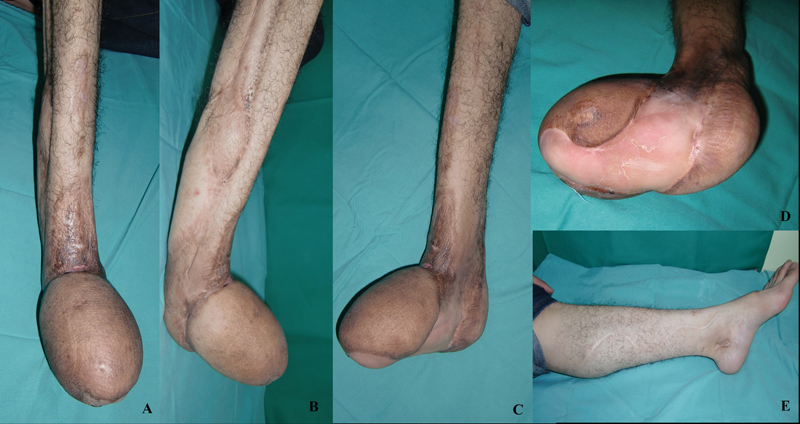
Four years after the lower limb salvage reconstruction. ( **A–D** ) No ischemia, contracture, and muscle atrophy noted over the right limb. ( **E** ) The donor site over the contralateral limb.

Cross-leg flaps are still valuable and reliable for reconstructing complex lower-limb wounds. It enables the reconstruction of limbs that would otherwise be unsalvageable. Taylor et al 11 first proposed cross-leg pedicled flaps in 1979 as a salvage attempt to restore a major defect after the recipient's vessels in the damaged leg spasmed, hindering microvascular anastomosis. Manrique et al advocate utilizing cross-leg free flaps for limbs with greater and more distal soft tissue lesions and one or no-vessel runoff. 12 Albeit our patient underwent transmetatarsal amputation, as much metatarsal length as possible was kept preserving the ankle's dorsiflexion and preventing equines deformity. To maximize the function of transmetatarsal bone, covering the bone with a flap rather than shortening it is crucial for this young patient. To go a step further, we performed a cross-leg double compound free flap as an ultima ratio to salvage the foot. This flap provides a mobile soft tissue envelope with proper contouring of bone ends to absorb shear and direct forces. Cross leg free flap allows strategic tailoring of donor tissue size, relatively longer pedicle across the leg with the anastomotic site completely out of trauma zone. The principle of a cross-leg free flap is laudable, but such merits have to be compared with potential complications due to prolonged immobility. The importance of physiotherapy, especially the day after the flap division, anticoagulant for deep vein thrombosis, and the use of ripple mattresses to prevent bedsores cannot be overemphasized. Ultimately, our patient achieved functional recovery with the cross-leg chimeric free-flap reconstruction. He can ambulate, and the follow-up X-ray also shows a good recovery of the bone fracture ( Fig. 5 ).

The advent of microsurgical soft-tissue transfer has broadened the definition of lower-extremity salvage procedures while narrowing the amputation requirements. Although delayed revascularizations are still associated with severe morbidity, with enhanced resuscitative attempts and coordinated surgical efforts to save the limb, only a small percentage of patients will fulfill the amputation criteria. Saving a limb with an arterial injury is a race against time. However, every attempt should be taken to restore a functional lower limb and improve life quality.

## References

[1] Chapman J A Popliteal artery damage in closed injuries of the knee J Bone Joint Surg Br 1985 67 03 420 423 3997952 10.1302/0301-620X.67B3.3997952

[2] Drapanas T Hewitt R L Weichert R F III Smith A D Civilian vascular injuries: a critical appraisal of three decades of management Ann Surg 1970 172 03 351 360 4917999 10.1097/00000658-197009000-00005 PMC1397345

[3] Steele H L Singh A Vascular injury after occult knee dislocation presenting as compartment syndrome J Emerg Med 2012 42 03 271 274 19345052 10.1016/j.jemermed.2008.08.029

[4] Green N E Allen B L Vascular injuries associated with dislocation of the knee J Bone Joint Surg Am 1977 59 02 236 239 845209

[5] Azouz S M Castel N A Vijayasekaran A Rebecca A M Lettieri S C Lower-limb reconstruction with chimeric flaps: the quad flap Microsurgery 2019 39 02 182 187 29737002 10.1002/micr.30335

[6] Yan H Zhao B Kolkin J The management of lower extremity multilevel arterial injuries: a 10-year experience PLoS One 2015 10 03 e0121769 25793506 10.1371/journal.pone.0121769 PMC4368051

[7] Perkins Z B Yet B Glasgow S Meta-analysis of prognostic factors for amputation following surgical repair of lower extremity vascular trauma Br J Surg 2015 102 05 436 450 25706113 10.1002/bjs.9689

[8] Baghi I Herfatkar M R Shokrgozar L Poor-Rasuli Z Aghajani F Assessment of vascular injuries and reconstruction Trauma Mon 2015 20 04 e30469 26839869 10.5812/traumamon.30469 PMC4727477

[9] Kohli A Singh G Management of extremity vascular trauma: Jammu experience Asian Cardiovasc Thorac Ann 2008 16 03 212 214 18515670 10.1177/021849230801600307

[10] Moini M Takyar M A Rasouli M R Revascularisation later than 24 h after popliteal artery trauma: is it worthwhile? Injury 2007 38 09 1098 1101 17697677 10.1016/j.injury.2007.05.001

[11] Taylor G I Townsend P Corlett R Superiority of the deep circumflex iliac vessels as the supply for free groin flaps Plast Reconstr Surg 1979 64 05 595 604 388478

[12] Manrique O J Bishop S N Ciudad P Lower extremity limb salvage with cross leg pedicle flap, cross leg free flap, and cross leg vascular cable bridge flap J Reconstr Microsurg 2018 34 07 522 529 29768632 10.1055/s-0038-1641712

